# Prolonged Waking and Recovery Sleep Affect the Serum MicroRNA Expression Profile in Humans

**DOI:** 10.3390/clockssleep1010008

**Published:** 2018-11-22

**Authors:** Susanne Weigend, Sebastian C. Holst, Josefine Meier, Matthias Brock, Malcolm Kohler, Hans-Peter Landolt

**Affiliations:** 1 Institute of Pharmacology and Toxicology, University of Zürich, 8057 Zürich, Switzerland; 2 Sleep and Health Zürich (SHZ), University of Zürich, 8057 Zürich, Switzerland; 3 Department of Pneumology, University Hospital Zürich, 8091 Zürich, Switzerland

**Keywords:** sleep loss, transcriptional regulators, miR-30c, biomarker, aging

## Abstract

MicroRNAs (miRNAs) are small, abundant, non-coding RNA fragments that regulate gene expression and silencing at the post-transcriptional level. The miRNAs each control various downstream targets and play established roles in different biological processes. Given that miRNAs were recently proposed to contribute to the molecular control of sleep–wake regulation in animal models and narcoleptic patients, we investigated the impact of acute sleep deprivation on blood miRNA expression in healthy adult men of two different age groups. Twenty-two young (mean age: 24 ± 3 years) and nine older (65 ± 1 years) volunteers completed a controlled in-lab study, consisting of 8 h baseline sleep, followed by 40 h of extended wakefulness, and a 10-h recovery sleep opportunity. At the same circadian time in all three conditions (at 4:23 p.m. ± 23 min), qPCR expression profiling of 86 miRNAs was performed in blood serum. Thirteen different miRNAs could be reliably quantified and were analyzed using mixed-model ANOVAs. It was found that miR-30c and miR-127 were reliably affected by previous sleep and wakefulness, such that expression of these miRNAs was upregulated after extended wakefulness and normalized after recovery sleep. Together with previous findings in narcolepsy patients, our preliminary data indicate that miR-30c and its target proteins may provide a biomarker of elevated sleep debt in humans.

## 1. Introduction

MicroRNAs (miRNAs) are non-protein coding RNA sequences proposed to function as guide-molecules for messenger RNA (mRNA) stability and translation in many biological and pathological processes [1]. The miRNAs are the dominating class of small non-coding RNAs and abundant in most somatic tissues. They consist of ~22 nucleotides in length and are produced by the so-called Drosha and Dicer RNase III proteins. Many miRNAs destabilize mRNA and thereby reduce protein levels [2], yet some miRNAs were also shown to promote mRNA stability and enhance translational processes under certain conditions [3,4]. In the central nervous system (CNS), miRNAs control distinct neuronal processes, such as brain morphogenesis, transcription of neuronal genes, and cell differentiation processes in neurons [5].

Emerging evidence suggests that miRNAs are important regulators of biological mechanisms relevant for the general aging processes of the brain and the vasculature, such as cell proliferation, apoptosis, and metabolism [6]. Indeed, studies in the nematode *C. elegans* revealed that miRNAs are genetic biomarkers of aging [7]. Consistent with the fact that miRNAs and genetic pathways of normal aging are highly conserved across species [8,9,10,11,12,13], a strong association between the circulating miRNA profile and aging was also found in humans [14,15]. In addition, circulating miRNAs were recently suggested as potential biomarkers of longevity [16].

A new line of research has recently linked miRNAs with sleep–wake regulation. More specifically, studies in Drosophila suggested that distinct miRNAs, particularly let-7, control the structure and amount of sleep, in addition to the sleep–wake dependent (i.e., homeostatic) aspect of sleep–wake regulation [17]. Sleep deprivation is the most pronounced physiological challenge of sleep homeostasis and drastically alters the blood proteome in animals and humans [18,19,20]. Recovery sleep after sleep deprivation is reliably and predictably more intense—as measured by electroencephalographic (EEG) slow-wave (SWA) or delta activity in non-rapid-eye-movement (NREM) sleep (EEG spectral power in the 0.75–4.5 Hz band)—and less fragmented when compared to baseline sleep [21,22,23]). In rats and mice, sleep deprivation and sleep fragmentation increased (miR-410, -212, -29c, -29b-2, and -708) and decreased (let-7e, miR-137, -22, -219-2, and -99a) miRNA expression in brain and adipose tissues [24,25,26]. Furthermore, injections of a miRNA-132 mimetic (preMIR-132) and specific inhibitors of miR-138, let-7b, and miR-125a (antiMIRs) into rat cerebral cortex differentially altered sleep. More specifically, antiMIR-138 reduced sleep and NREM EEG delta power, whereas injection of preMIR-132 decreased NREM sleep duration, yet enhanced NREM sleep SWA and rapid-eye-movement (REM) sleep duration [27,28].

In animals and humans, sleep typically becomes more superficial and fragmented with increasing age [29,30,31,32]. Furthermore, when neurophysiologic and neurobehavioral markers of sleep homeostasis are considered, older people are typically less vulnerable to sleep deprivation than young people [33,34,35]. The molecular underpinnings of these age-related changes in sleep–wake physiology are currently unknown.

No study has yet examined sleep deprivation-induced changes in the human blood miRNA profile. Nevertheless, it was recently reported that sleepy patients suffering from narcolepsy and other central disorders of hypersomnolence exhibit altered levels of specific miRNAs in blood plasma when compared to healthy controls [36]. In this exploratory investigation, we addressed the hypothesis that acute sleep deprivation and recovery sleep may differently affect blood miRNA levels in healthy men of young (24 ± 3 years) and older (65 ± 1 years) age.

## 2. Results

### Impact of Sleep Deprivation and Recovery Sleep on miR-30c and miR-127 Expression in Blood Serum

**Figure 1:**
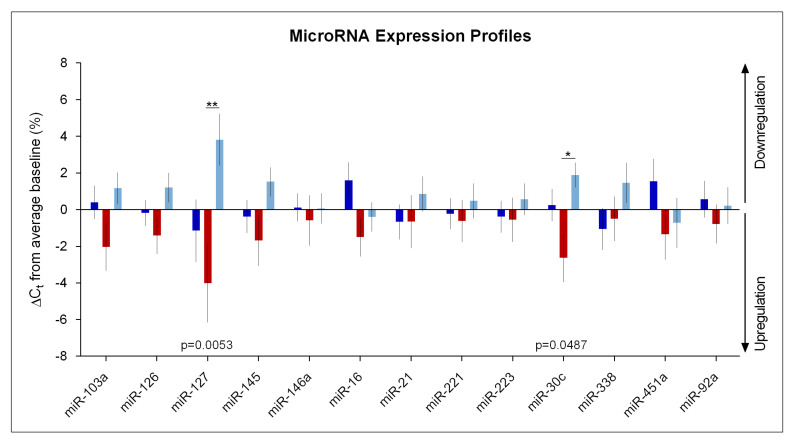
MicroRNA expression profiles in human blood serum. Relative expression (ΔC_t_ values) of thirteen miRNAs in baseline (‘BL’, dark blue), sleep deprivation (‘SD’, red), and recovery (‘REC’, light blue) conditions were expressed as a percentage change from the overall average in the entire study sample (*n* = 31). Positive values indicate downregulation, negative values indicate upregulation. The *p*-values represent a significant main effect of ‘condition’ of a repeated-measures, mixed-model ANOVA with the between-subject factors ‘condition’ (BL, SD, REC) and ‘age group’ (young, older). Asterisks indicate significant differences between conditions (* *p* < 0.05; ** *p* < 0.01). Values and error bars represent mean and standard error of the mean (SEM).

Of the 86 target miRNAs (Table S1), 13 could be repeatedly measured and reliably quantified (Table 1). The possible effects of sleep deprivation and age on these miRNAs were statistically analyzed. The analysis of the entire study sample revealed that miR-30c and miR-127 were significantly affected by the sleep condition (‘condition’, *p*_FDRcorrected_ < 0.05; Table 1). Both these miRNA species were upregulated by sleep deprivation and downregulated following recovery sleep (Figure 1). No significant main effect of ‘age’ nor a significant ‘condition’ x ‘age’ interaction were detected for any of the miRNAs analyzed (Table 1).

**Figure 2:**
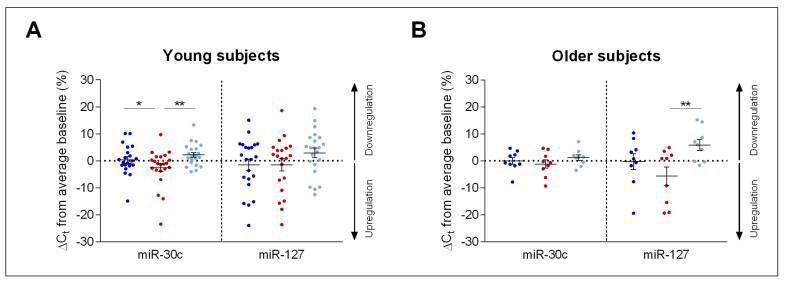
Expression profiles of miR-30c and miR-127 in young and older individuals. Individual ΔC_t_ values of miR-30c and miR-127 are displayed in percent change from average baseline in young ((**A**), *n* = 22) and older ((**B**), *n* = 9) healthy male volunteers. Baseline (dark blue), sleep deprivation (red) and recovery (light blue) conditions are shown. Asterisks indicate significant differences between conditions (* *p* < 0.05; ** *p* < 0.01). Error bars represent mean and standard error of the mean (SEM).

To explore the sleep–wake dependent changes in miR-30c and miR-127 expression in more detail, the individual data of all study participants across the three experimental conditions were plotted (Figure 2). In young adults, expression of miR-30c was upregulated by the night without sleep and normalized after recovery sleep (1-way ANOVA with factor ‘condition’: F_2,41_ = 4.26, *p* < 0.03). Inspection of Figure 2A demonstrates that 12 of the 22 young study participants showed this overall pattern of sleep–wake dependent changes in miR-30c expression. By contrast, the factor ‘condition’ was not significant when only the older study participants were considered (F_2,16_ = 0.95, *p* > 0.4; Figure 2B).

In the older age group, however, miR-127 expression was affected by experimental ‘condition’ (1-way ANOVA: F_2,16_ = 4.37, *p* < 0.04). While sleep deprivation appeared to upregulate miR-127 without reaching statistical significance (*p* = 0.15, Tukey-Kramer post-hoc test), recovery sleep was associated with a pronounced downregulation of this miRNA in older men (Figure 2B). In contrast to miR-30c, the sleep–wake related changes in miR-127 were not significant in the young age group (F_2,41_ = 1.91, *p* = 0.16).

## 3. Discussion

This study provides the first experimental evidence in humans that the expression patterns of distinct miRNAs in blood serum are upregulated by acute total sleep deprivation and normalized following recovery sleep. Intriguingly, one of the affected miRNA species, miR-30c, was previously reported to be elevated in blood plasma in patients suffering from narcolepsy type-1 when compared to healthy controls [36]. Narcolepsy patients suffer from severe daytime sleepiness, and their state has been compared to healthy people who have undergone 1–2 nights of total sleep deprivation [37,38]. Together, these observations indicate that miR-30c and its target proteins may be potential biomarkers of an elevated sleep debt.

It has been found that miR-30c is highly expressed in primary cardiomyocytes and modulates cellular hypertrophy [39]. It has been suggested that the upregulation of miR-30c expression observed in narcolepsy type-1 [36,40] contributes to altered night-time blood pressure regulation and heart rate response, and possibly result in increased cardiovascular risk in these patients [41,42]. Heart rate and cardiovascular functions are also affected by acute total sleep deprivation (for review see Mullington et al., 2009 [43]). It may be tempting to speculate that the sleep loss-induced changes in miR-30c expression provide a molecular link between sleep deprivation and its consequences on peripheral nervous system regulation.

Apart from cardiovascular functions, miR-30c and its target genes are involved in a wide range of pathways that play important roles in sleep–wake regulation (see supplementary Table S2). Among these, serotonergic, dopaminergic, and histaminergic signaling is of particular interest because their relevance in sleep–wake neurochemistry is well established (Holst and Landolt, 2018 for recent review [44]). Several metabolomics studies found a sleep loss-induced upregulation of serotonin and serotonin-related genes [45,46]. Thus, it may be speculated that miR-30c could provide a possible transcriptional link between sleep deprivation and sleep loss-induced metabolic changes.

Age-related differences in the effects of sleep loss on neurophysiological and neurobehavioral markers of elevated sleep pressure are well established [33,34,35]. Against this background, it is interesting to note that sleep deprivation and recovery sleep altered miR-30c in the young age group yet had no effects in the older age group. The mean age of the patients and controls studied by Holm et al. was 27.8 years [36,40], which was very similar to the age range of the young age group in our study (24.5 years). It may be speculated that older age is associated with an attenuated response of miR-30c expression to sleep deprivation. On the other hand, it is also possible that the current study had insufficient statistical power to detect significant sleep–wake dependent changes in miR-30c in the smaller, older age group. The lack of a prior study quantifying miRNA expression as a function of sleep deprivation and recovery sleep in humans rendered an a-priory power analysis unfeasible. Hence, the possibility of insufficient statistical power cannot be excluded.

Conversely, the statistical analyses demonstrated that miR-127 expression was altered by wakefulness and sleep in the older group, suggesting that this sample was large enough to detect statistically significant changes. miR-127 is primarily involved in cancer pathologies by regulating cell proliferation and senescence through the oncogene BCL6 [47]. Cellular senescence is an important stress response, and several omics studies found an effect of sleep deprivation and recovery sleep on specific cancer and senescence-related genes [48,49,50].

In conclusion, consistent with prior animal experiments suggesting distinct roles for miRNAs in sleep–wake regulation and aging [14,15,17,24,25,26,27,28], we here report for the first time that wakefulness and sleep affect the blood miRNA expression profile in young and older men. Given the rather small study sample, the reported results need to be considered preliminary. Nevertheless, future studies investigating the roles of miRNAs in sleep–wake regulation are clearly warranted. By scrutinizing the target pathways of distinct miRNAs, new light may be shed on the molecular processes underlying the fine-tuned physiological regulation of wakefulness and sleep and their age-related changes in health and disease.

## 4. Materials and Methods

### 4.1. Study Participants

The study protocol and all procedures were approved by the ethics committee of the Canton of Zürich for research on human subjects (ethics approval number: 2015-0424, 2 December 2015) and complied with the principles in the Declaration of Helsinki. Prior to the experiments, subjects provided written informed consent, and they received financial compensation for their participation at completion of the study.

After the extensive exclusion of subjects with medical, neurological, or psychiatric diseases, twenty-two young and nine older healthy men completed a 1-week experimental protocol (see below). All participants were non-smokers, in good health, and had not taken any medications or consumed illicit drugs within 2 months prior to the study. They did not cross time zones or work night shifts during the last 3 months before participation. Based on polysomnographic screening in the sleep laboratory prior to enrollment into the study, subjects with sleep disturbances, such as sleep apnea, sleep efficiency < 75%, or a periodic leg movement in sleep (PLMS) index of 5 or more per hour of sleep were excluded from participation. Table 2 summarizes lifestyle and demographic characteristics of the study sample as assessed by validated questionnaires. The two age groups displayed significant differences in caffeine consumption, chronotype/diurnal preference, trait anxiety, and personality traits. Nevertheless, all values were considered normal or in the moderate range (caffeine consumption [51]; diurnal preference [52]; trait anxiety [53]; Eysenck personality questionnaire [54]). Both age groups reported on average a habitual sleep duration of 7–8 h (Table 2).

### 4.2. Pre-Experimental Procedure and Experimental Protocol

Two weeks prior to the study, participants were asked to refrain from all sources of caffeine and alcohol, and to wear a wrist activity monitor on the non-dominant arm to guarantee a regular 8-h night-time sleep schedule, corresponding approximately to the participants’ habitual sleep times. In addition, daily log-books verified compliance with the pre-study instructions. To confirm participants’ abstinence, caffeine and ethanol concentrations in saliva and breath were tested upon entering the laboratory.

**Figure 3:**
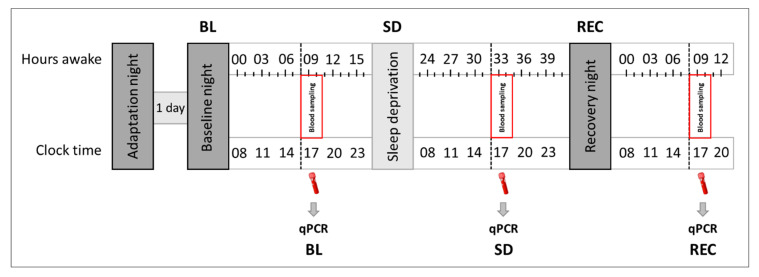
Experimental protocol. Following 8-h adaptation and baseline nights, all study participants were kept awake for 40 h, followed by a 10-h recovery night. In baseline (‘BL’), sleep deprivation (‘SD’), and recovery (‘REC’) conditions, blood samples for the quantification of plasma miRNA levels were drawn at the same circadian time (at 4:23 p.m. ± 23 min). During wakefulness, participants completed cognitive test sessions every three hours and were constantly supervised throughout the protocol.

Under constant supervision, all subjects completed a sleep deprivation protocol, consisting of 8-h adaptation and baseline nights (time in bed: 11:00 p.m.–7:00 a.m.), followed by 40 h of continuous wakefulness, and terminated by a 10-h recovery night (Figure 3). Every 3 h, participants underwent a test battery consisting of vigilance (Psychomotor Vigilance Task (PVT) [59]), sleepiness (Karolinska Sleepiness Scale (KSS) [60]), tiredness symptoms (Tiredness Symptoms Scale (TSS) [61]), and affective state (Visual Analogue Scales (VAS) [62]) testing. These data will be reported elsewhere. At the same circadian time (at 4:23 p.m. ± 23 min) in ‘baseline’, ‘sleep deprivation’, and ‘recovery’ conditions, all subjects provided a blood sample to analyze and quantify circulating miRNA concentrations in blood serum (within-subjects design).

### 4.3. Assessment of miRNA

Real-time quantitative polymerase chain reaction (qPCR) expression profiling of 86 miRNAs was performed with RNA from blood serum samples of all subjects using the miScript miRNA PCR Array (QIAGEN Inc., Germantown, MD, USA). The 86 target miRNAs are listed in Supplementary Table S1. This array tested for the expression of various miRNAs previously suggested to control pathways regulating sleep and waking [17,24,25,26,27,28]. For each sample, two independent runs were performed to obtain duplicate values.

### 4.4. Isolation of miRNA

Total RNA including miRNAs was purified from human serum samples using the miRNeasy Isolation Kit (QIAGEN Inc., Germantown, MD, USA). The isolation procedure was applied and conducted as recommended by the manufacturer.

### 4.5. Quantification of Mature miRNAs

The miScript miRNA PCR Array was carried out according to the manufacturer’s instructions. Quantification of complementary DNA (cDNA) was performed using qPCR with the StepOnePlus system (Applied Biosystem, Thermo Fisher Scientific, Waltham, MA, USA). Specific amplification of the miRNAs of interest was confirmed by melting curve analysis. Differential miRNA expression was calculated with the threshold cycle (C_t_) method [63]. miRNA expression levels were expressed as normalized Δc_t_ values in percentage change from the average baseline (average of all three conditions (baseline, sleep deprivation, recovery) in each subject). Higher Δc_t_ values indicate a downregulation and lower Δc_t_ values indicate an upregulation of miRNA expression compared to the average of all three conditions.

### 4.6. Hemolysis in Human Blood Serum Samples

It is described that the expression of some miRNAs can be inhibited or diminished through hemolysis [64]. Therefore, we tested all samples for hemolysis, by quantifying the serum levels of miR-451a (in duplicates), a miRNA that is highly expressed in erythrocytes. To avoid a possible influence of sleep deprivation on miR-451a alone, the levels were normalized to stably expressed miR-23a-3p [65]. Supplementary Figure S1 displays the averaged C_t_ values of miR-451a minus the C_t_ values of miR-23a-3p for each condition (mean ± SEM). All values were similar and did not differ between the three conditions (*p*_all_ > 0.3), demonstrating that absence of hemolysis in the samples can be assumed [66].

### 4.7. Identification of Genes and Pathways

Two databases, namely miRDB and TargetScanHuman v7.2 [67,68,69], were used to identify the target genes of those miRNAs, which were found altered by sleep deprivation and recovery sleep (miR-30c-5p, miR-127-3p). The biological pathways accessed and possibly regulated by the differentially expressed miRNAs were subsequently examined by a pathway analysis using the PANTHER Gene Ontology classification system with default settings [70,71].

### 4.8. Statistical Analyses

All statistical analyses were performed with SAS 9.4 software (SAS Institute, Cary, NC, USA). Mixed-effect repeated-measures analyses of variance (ANOVA) models included the factors ‘condition’ (baseline, sleep deprivation, recovery) and ‘age group’ (young, older) and their interaction. Correction for multiple comparisons in the mixed-model ANOVAs involved false discovery rate (FDR) calculations (α < 0.05). Comparisons of least squared means of conditions in ANOVA analyses were post-hoc corrected (Tukey-Kramer correction: α < 0.05). If not stated otherwise, only significant results are shown. Mean values reported in Tables and Figures represent mean ± standard error of the mean (SEM) expressing between-subject variability.

## Figures and Tables

**Table 1:** Statistical analysis of thirteen reliably measured miRNAs with 2-way mixed-model ANOVA for repeated measures, with the within-subjects factor ‘condition’ (baseline (BL), sleep deprivation (SD), recovery (REC)) and the between-subjects factor ‘age’ (young, older) and their interaction (*n* = 31). Corrected *p*-values represent false discovery rate (FDR) correction for multiple comparisons. Significant effects are highlighted in bold.

miRNA	Factor	F-Value	*p*-Value (Uncorrected)	*p*-Value (Corrected)
miR103a-3p	Condition	F_2,57_ = 2.29	0.07	0.11
	Age Condition x Age	F_1,57_ = 0.00 F_2,57_ = 0.57	0.89 0.49	0.99 0.57
miR126-3p	Condition	F_2,57_ = 0.82	0.23	0.45
	Age Condition x Age	F_1,57_ = 0.02 F_2,57_ = 1.25	0.65 0.11	0.89 0.29
**miR127-3p**	**Condition**	**F_2,57_ = 5.76**	**0.0001**	**0.005**
	Age Condition x Age	F_1,57_ = 0.00 F_2,57_ = 0.97	0.91 0.24	0.97 0.38
miR145-5p	Condition	F_2,57_ = 1.10	0.23	0.34
	Age Condition x Age	F_1,57_ = 0.01 F_2,57_ = 0.46	0.82 0.51	0.91 0.64
miR146a-5p	Condition	F_2,57_ = 0.04	0.90	0.96
	Age Condition x Age	F_1,57_ = 0.02 F_2,57_ = 0.18	0.92 0.73	0.89 0.84
miR16-5p	Condition	F_2,57_ = 1.58	0.07	0.22
	Age Condition x Age	F_1,57_ = 0.01 F_2,57_ = 2.24	0.82 0.08	0.91 0.10
miR21-5p	Condition	F_2,57_ = 0.56	0.31	0.58
	Age Condition x Age	F_1,57_ = 0.05 F_2,57_ = 1.00	0.80 0.25	0.82 0.37
miR221-3p	Condition	F_2,57_ = 0.64	0.45	0.53
	Age Condition x Age	F_1,57_ = 0.00 F_2,57_ = 0.59	0.93 0.30	0.97 0.56
miR223-3p	Condition	F_2,57_ = 0.09	0.87	0.92
	Age Condition x Age	F_1,57_ = 0.01 F_2,57_ = 1.90	0.83 0.09	0.90 0.16
**miR30c-5p**	**Condition**	**F_2,57_ = 3.18**	**0.003**	**0.05**
	Age Condition x Age	F_1,57_ = 0.01 F_2,57_ = 0.43	0.87 0.39	0.92 0.65
miR338-5p	Condition	F_2,57_ = 1.63	0.07	0.21
	Age Condition x Age	F_1,57_ = 0.01 F_2,57_ = 0.55	0.84 0.31	0.91 0.58
miR451a	Condition	F_1,57_ = 1.01	0.25	0.37
	Age Condition x Age	F_2,57_ = 0.03 F_2,57_ = 2.24	0.85 0.08	0.87 0.10
miR92a-3p	Condition	F_1,57_ = 0.06	0.91	0.95
	Age Condition x Age	F_2,57_ = 0.01 F_2,57_ = 1.73	0.90 0.10	0.94 0.19

**Table 2:** Demographic data of study participants.

Variable	Young Age Group (Mean ± SD)	Older Age Group (Mean ± SD)	*p*-Value
Sample size (n)	22	9	
Age (years)	24.45 ± 3.23	64.89 ± 1.17	
Body Mass Index (kg/m^2^)	23.19 ± 2.09	23.97 ± 2.62	0.22
Caffeine Consumption (mg/day)	112.27 ± 101.73	294.37 ± 3.06	0.0007
Alcohol consumption (drinks/week)	3.52 ± 3.26	3.06 ± 3.41	0.37
Daytime sleepiness (ESS)	5.68 ± 3.03	8.00 ± 3.71	0.06
Habitual sleep duration (min)	445.91 ± 6.63	438.33 ± 10.54	0.28
Pittsburgh sleep quality index (PSQI)	3.45 ± 0.32	3.22 ± 0.61	0.37
Diurnal preference (MEQ)	52.09 ± 9.29	61.00 ± 11.08	0.03
Trait anxiety (TAI)	30.71 ± 5.98	26.13 ± 6.96	0.04
Eysenck Personality Traits			
Psychoticism	3.64 ± 2.34	1.22 ± 1.09	0.0003
Extraversion	7.27 ± 2.73	7.11 ± 4.01	0.46
Neuroticism	2.05 ± 2.42	1.78 ± 2.17	0.38
Lie scale	3.45 ± 1.52	5.1 ± 2.83	0.03
Depression score (BDI)	3.14 ± 4.56	2.33 ± 2.41	0.26
Cognitive assessment (MoCA)	29.43 ± 0.73	28.8 ± 0.81	0.46

Thirty-one healthy male participants completed the study protocol and were grouped into twenty-two young (20–30 years) and nine older (60–70 years) participants. Validated German translations and versions of questionnaires were used to assess lifestyle and personality traits. Caffeine consumption was calculated based on the following amounts per serving: coffee: 100 mg; ceylon or green tea: 30 mg; cola drink: 40 mg (2 dL); energy drink: 80 mg (2 dL); chocolate: 50 mg (100 g). Diurnal preference: Horne-Östberg Morningsness-Eveningness Questionnaire [52]; daytime sleepiness: Epworth Sleepiness Scale [55]; depression score: Beck Depression Inventory [56]; personality traits: Eysenck Personality Questionnaire [54]; cognitive assessment: Montreal Cognitive Assessment [57]; trait anxiety: State-Trait Anxiety Inventory [53]; sleep quality: Pittsburgh Sleep Quality Index [58].

## Supplementary Materials

The following are available online at http://www.mdpi.com/2624-5175/1/1/8/s1, Figure S1: Hemolysis test for impurities, Table S1: List of tested miRNAs in miRNA PCR array, Table S2: Analysis of miRNA-targeted genes and pathways using miRDB and TargetScanHuman v7.2 (genes) and PANTHER Gene Ontology classification system (pathways).
