# Supplementary material for: Prolonged Waking and Recovery Sleep Affect the Serum MicroRNA Expression Profile in Humans
Source: Clocks Sleep. 2018 Nov 22;1(1):75–86. doi: 10.3390/clockssleep1010008 (PMC7509676; doi:10.3390/clockssleep1010008)
Supplement: Supplementary file 1 [file clockssleep-01-00008-s001.pdf]

Supplementary Material

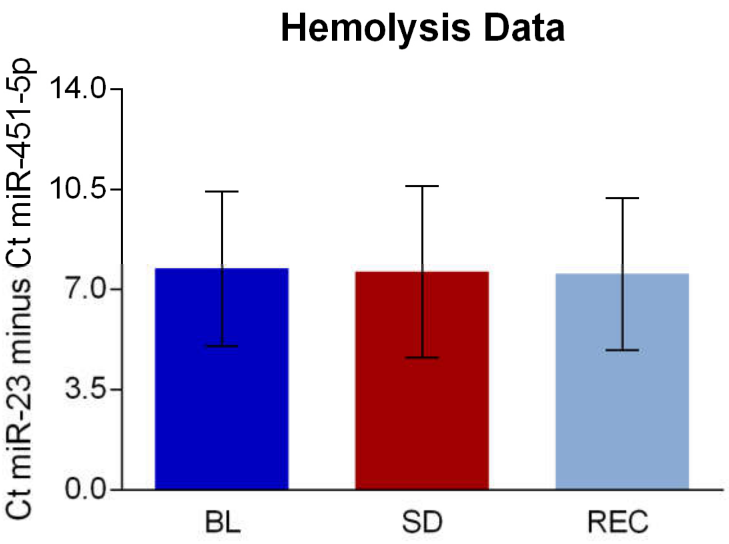

Figure S1. Hemolysis test for impurities. Levels of miR-451a-5p and miR-23a were analyzed and quantified by qPCR in each sample and respective duplicate. The C_t value of miR-451a-5p minus C_t value of miR-23a was calculated for each sample and means for each condition ('BL' = baseline, 'SD' = sleep deprivation, 'REC' = recovery) were plotted. Error bars represent SEM.

Table S1. List of tested miRNAs in miRNA PCR array.

well	miRNA	well	miRNA	well	miRNA
A1	miR-1-3p	C5	miR-155-5p	E9	miR-30d-5p
A2	miR-100-5p	C6	miR-15b-5p	E10	miR-31-5p
A3	miR-103a-3p	C7	miR-16-5p	E11	miR-323a-3p
A4	miR-106b-5p	C8	miR-17-5p	E12	miR-324-5p
A5	miR-107	C9	miR-181a-5p	F1	miR-331-5p
A6	miR10a-5p	C10	miR-182-5p	F2	miR-338-5p
A7	miR124-3p	C11	miR-183-5p	F3	miR-339-5p
A8	miR-125-5p	C12	miR-199a-3p	F4	miR-34a-5p
A9	miR-126-3p	D1	miR-203a-3p	F5	miR-34c-5p
A10	miR-127-3p	D2	miR-204-3p	F6	miR-365a-3p
A11	miR-128-3p	D3	miR-206	F7	miR-369-5p
A12	miR-129-3p	D4	miR-208a-3p	F8	miR-374b-5p
B1	miR-130b-3p	D5	miR-20a-5p	F9	miR-376b-5p
B2	miR-132-3p	D6	miR-20b-5p	F10	miR378a-3p
B3	miR-132-5p	D7	miR-210-3p	F11	miR-379-3p
B4	mir-133a-3p	D8	miR-214-3p	F12	miR-451a
B5	miR-133b	D9	miR-21-5p	G1	miR-487b-3p
B6	miR-134-5p	D10	miR-219a-2-3p	G2	miR-495-3p
B7	miR-137	D11	miR-219a-5p	G3	miR-505-5p
B8	miR-138-5p	D12	miR-221-3p	G4	miR-543
B9	miR-142-3p	E1	miR-223-3p	G5	miR-665
B10	miR-142-5p	E2	miR23b-3p	G6	miR-7-5p

B11	miR-143-3p	E3	miR-25-3p	G7	miR-92a-3p
B12	miR-145-5p	E4	miR-29a-3p	G8	miR-92b-3p
C1	miR-146a-5p	E5	miR-302d-3p	G9	miR-93-5p
C2	miR-146b-5p	E6	miR-30a-3p	G10	miR96-5p
C3	miR-152-3p	E7	miR-30b-5p	G11	miR98-5p
C4	miR-154-5p	E8	miR-30c-5p	G12	miR-99a-5p

Table S2. Analysis of miRNA-targeted genes and pathways using miRDB and TargetScanHuman v7.2 (genes) and PANTHER Gene Ontology classification system (pathways). Number of genes associated with the sleep regulated miRNAs is noted in the table. Pathways accessed by genes of both miR-30c (total # genes: 204, pathway hits: 94) and miR-127 (total # genes: 17, pathway hits: 7) are highlighted in blue.

**Pathway**	**miR-30c # genes**	**miR-127 # genes**
Sleep-wake regulated pathways:		
$5HT_1$ type receptor mediated signaling pathway	2	0
$5HT_2$ type receptor mediated signaling pathway	1	0
Alzheimer disease-amyloid secretase pathway	0	1
Alzheimer disease-presenilin pathway	3	1
Apoptosis signaling pathway	1	0
Cadherin signaling pathway	4	1
Dopamine receptor mediated signaling pathway	1	0
EGF receptor signaling pathway	1	0
FGF signaling pathway	1	0
GABA-B receptor II signaling	1	0
Gamma-aminobutyric acid synthesis	1	0
Gonadotropin-releasing hormone receptor pathway	2	0
Histamine H1 receptor mediated signaling pathway	1	0
Histamine H2 receptor mediated signaling pathway	1	0
Inflammation mediated by chemokine and cytokine signaling pathway	3	0
Integrin signaling pathway	4	1
Interleukin signaling pathway	1	0
Oxytocin receptor mediated signaling pathway	1	0
Parkinson disease	2	0
PI3 kinase pathway	1	0
Wnt signaling pathway	7	1
Non sleep-wake regulated pathways:		
$5HT_4$ type receptor mediated signaling pathway	1	0
Alpha adrenergic receptor signaling pathway	1	0
Angiogenesis	3	1
Angiotensin II-stimulated signaling through G proteins and beta-arrestin	1	0
Beta1 adrenergic receptor signaling pathway	1	0
Beta2 adrenergic receptor signaling pathway	1	0
Beta3 adrenergic receptor signaling pathway	1	0
CCKR signaling map	1	0
Cell cycle	1	0
Cortocotropin releasing factor receptor signaling pathway	1	0
Cytoskeletal regulation by Rho GTPase	3	0

Endogenous cannabinoid signaling	1	0
Enkephalin release	2	0
Hedgehog signaling pathway	1	0
Heterotrimeric G-protein signaling pathway-Gi alpha and Gs alpha mediated pathway	4	0
Heterotrimeric G-protein signaling pathway-Gq alpha and Go alpha mediated pathway	2	0
Heterotrimeric G-protein signaling pathway-rod outer segment phototransduction	1	0
Huntington disease	3	0
Insulin/IGF pathway-mitogen activated protein kinase kinase/MAP kinase cascade	1	0
Metabotropic glutamate receptor group II pathway	2	0
Metabotropic glutamate receptor group III pathway	2	0
Muscarinic acetylcholine receptor 1 and 3 signaling pathway	1	0
Muscarinic acetylcholine receptor 2 and 4 signaling pathway	2	0
N-acetylglucosamine metabolism	1	0
Nicotinic acetylcholine receptor signaling pathway	1	0
Opioid prodynorphin pathway	2	0
Opioid proenkephalin pathway	2	0
Opioid proopiomelanocortin pathway	2	0
PDGF signaling pathway	1	0
Thyrotropin-releasing hormone receptor signaling pathway	1	0
Ubiquitin proteasome pathway	2	0
